# Deep neural network-estimated age using optical coherence tomography predicts mortality

**DOI:** 10.1007/s11357-023-00920-4

**Published:** 2023-09-21

**Authors:** Ruiye Chen, Shiran Zhang, Guankai Peng, Wei Meng, Grace Borchert, Wei Wang, Zhen Yu, Huan Liao, Zongyuan Ge, Mingguang He, Zhuoting Zhu

**Affiliations:** 1 Centre for Eye Research Australia; Ophthalmology, University of Melbourne, Melbourne, Australia; 2 Ophthalmology, Department of Surgery, University of Melbourne, Melbourne, Australia; 3 State Key Laboratory of Ophthalmology, Zhongshan Ophthalmic Center, Sun Yat-Sen University, Guangzhou, China; 4 Guangzhou Vision Tech Medical Technology Co., Ltd, GuangZhou, China; 5 Central Clinical School, Monash University, Melbourne, Australia; 6 Epigenetics and Neural Plasticity Laboratory, Florey Institute of Neuroscience and Mental Health, University of Melbourne, Melbourne, Australia; 7 Faculty of IT, Monash University, Melbourne, Australia; 8 Monash Medical AI, Monash University, Melbourne, Australia

**Keywords:** Optical coherence tomography, Deep neural network, Age gap, Mortality

## Abstract

The concept of biological age has emerged as a measurement that reflects physiological and functional decline with ageing. Here we aimed to develop a deep neural network (DNN) model that predicts biological age from optical coherence tomography (OCT). A total of 84,753 high-quality OCT images from 53,159 individuals in the UK Biobank were included, among which 12,631 3D-OCT images from 8,541 participants without any reported medical conditions at baseline were used to develop an age prediction model. For the remaining 44,618 participants, OCT age gap, the difference between the OCT-predicted age and chronological age, was calculated for each participant. Cox regression models assessed the association between OCT age gap and mortality. The DNN model predicted age with a mean absolute error of 3.27 years and showed a strong correlation of 0.85 with chronological age. After a median follow-up of 11.0 years (IQR 10.9–11.1 years), 2,429 deaths (5.44%) were recorded. For each 5-year increase in OCT age gap, there was an 8% increased mortality risk (hazard ratio [HR] = 1.08, CI:1.02–1.13, *P* = 0.004). Compared with an OCT age gap within ± 4 years, OCT age gap less than minus 4 years was associated with a 16% decreased mortality risk (HR = 0.84, CI: 0.75–0.94, *P* = 0.002) and OCT age gap more than 4 years showed an 18% increased risk of death incidence (HR = 1.18, CI: 1.02–1.37, *P* = 0.026). OCT imaging could serve as an ageing biomarker to predict biological age with high accuracy and the OCT age gap, defined as the difference between the OCT-predicted age and chronological age, can be used as a marker of the risk of mortality.

**Supplementary Information:**

The online version contains supplementary material available at 10.1007/s11357-023-00920-4.

## Introduction

Chronological age refers to the number of years a person has been alive. It is a major risk factor for frailty, age-related morbidity and mortality [1]. However, there is considerable variability in health outcomes among individuals with the same chronological age, suggesting a heterogeneous rate of ageing [2]. Based on this, the concept of biological age has emerged as a measurement that reflects physiological and functional decline with ageing [3, 4]. Accurate quantification of biological age is crucial for risk stratification of individuals with accelerated ageing and exploring anti-ageing interventions, reducing the burden on public health. Several biomarkers have been proposed, among which retinal age derived from retinal images enables a non-invasive, quick and easy way of quantifying biological age [5]. Retinal age gap, the difference between retinal and chronological age has been verified to be strong predictive markers for all-cause mortality and age-related morbidities, such as cardiovascular diseases, neurodegenerative diseases, and kidney failure. [6–10]

Recent advances in imaging technology, particularly optical coherence tomography (OCT), are widely applied to visualize the retina [11–13]. Compared with two-dimensional fundus images mainly focusing on the vessels, OCT uses light to capture 2D and 3D images up to a resolution of a micrometer (μm), enabling better visualization of subtle changes related to ageing [14]. Furthermore, OCT provides visualization of not only the vasculature but also the neural tissue [13]. Emerging evidence has suggested that OCT imaging provides insight into ageing. Specifically, structural parameters such as the peripapillary retinal nerve fiber layer (RNFL) thickness and macula thickness were negatively associated with age [15, 16]. Vascular parameters such as choriocapillaris diameters tended to decrease with age [14, 17]. Therefore, we hypothesized that OCT images could provide a more comprehensive fingerprints for age prediction compared to fundus photographs.

Previously, two studies investigated age prediction based on OCT imaging. Shigueoka et al. implemented a deep learning (DL) model using B-scans from 278 participants and the predicted age was strongly correlated with chronological age with mean absolute error (MAE) of 5.82 years. [18] Another DL model trained on 3134 participants aged from 20 to 91 years achieved an average MAE of 5.78 years. [19] To the best of our knowledge, few studies have investigated whether OCT images could be used to predict biological age. In this study, we aimed to develop a biological age estimation model based on OCT scans in a healthy population and explore the predictive value of the OCT age gap, defined as the difference between OCT-predicted and chronological age, for all-cause mortality.

## Methods

### Study population

The UK Biobank is a large-scale, population-based cohort with more than 500, 000 participants between 40 to 69 years old and recruited from 2006 to 2010. All participants were asked to complete baseline assessment, which included healthcare questionnaires from digital screens and comprehensive physical examinations. Sampling of blood, urine and saliva were also completed at baseline. Health events during the follow-up period were collected through data linkage to hospitals and death registers. A detailed study protocol has been previously described. [20]

This study was reviewed and approved by the National Information Governance Board for Health and Social Care and the NHS North West Multicenter Research Ethics Committee (11/NW/0382) and the UK Biobank consortium (application no. 94372). The study was conducted according to the Declaration of Helsinki, with informed consent obtained from all participants.

### Spectral-domain optical coherence tomography imaging protocol

Ophthalmic examinations were conducted between 2009 and 2010 and included physical measurements (visual acuity, autorefraction and intraocular pressure) and ocular imaging (retinal fundus and OCT). The OCT images were collected under mesopic conditions without pupillary dilation using the Topcon 3D OCT 1000 Mk2 (Topcon Corp., Tokyo, Japan). This was achieved using a 3D macular volume scan of 6 mm × 6 mm pattern with 512 A-scans by 128 B-scans. A very small galvanometer was used and each image was acquired over a duration of 3.7 s. A total of 68,525 participants took OCT images in the UK Biobank study.

### Eligible criteria for OCT images

There were 84,753 OCT images of a high-quality from 53,159 individuals. The quality control processes used have been thoroughly described in previous studies [21, 22]. In brief, version 1.6.1.1 of the Topcon Advanced Boundary Segmentation (TABS) algorithm has been used to generate several segmentation indicators to identify poor scan quality or segmentation failures [23]. These indicators included image quality score, an inner limiting membrane indicator, a validity count, and motion indicators [21]. The OCT imaging quality was scaled from 0 to 100, with a higher score indicating a better image quality. Images were excluded if an image quality scored less than 45 (the maximum score was 44), poor centration certainty, or poor segmentation certainty (defined as the poorest 20% of images based on each of the segmentation indicators).

### Deep neural network model for age prediction

To build the age prediction model, the OCT images of both eyes where available were used. Consistent with previous studies,[6, 24] a total of 12,631 3D-OCT images of 8,541 healthy participants without reported medical conditions at baseline were used to develop the age prediction model. For training and validation, 7,687 (90%) individuals were randomly selected and five-fold cross-validation was used for internal validation. We constructed the ResNet-3D network based on the ResNet model developed by a previous study [25]. ResNet is a deep convolutional neural network used for image classification tasks. In this study, the ResNet-3D algorithm was designed based on the ResNet model and used 18 layers for image training. The input shapes included the clip length, image height, image width, and number of channels, which were defined as 128, 256, 256, and 1, respectively. The number of outputs at the final linear layer were set to one, and the regularizer factor was set to the default value of 1e-4. To improve the performance of the neural network, we used stochastic gradient descent (SGD) with momentum that equaled to 0.9. The selection of candidate DL models was based on the mean absolute error (MAE) in the validation set. For testing, the remaining 854 (10%) participants were used. The MAE and correlation between predicted retinal age and chronological age were then calculated to assess the performance of the model.

### OCT age gap definition

For the remaining 44,618 participants, OCT-predicted age was generated for each participant. Images of the right eye (if available) were used to calculate OCT-predicted age, and if the right eye images were not available, then the left eye images were used. The difference between the OCT-predicted age and chronological age was defined as OCT age gap.

### Mortality ascertainment

Mortality data was accessed through the data linkage to hospitals and the national mortality registry. The follow-up period was the time from image acquisition to death, lost to follow-up, or the last follow-up date (28^th^ April 2021), whichever came earliest.

### Covariates

Similarly with our previous study [5], potential confounding factors associated with mortality were adjusted for and included age (continuous, years), gender (male or female), race (white or non-white), townsend deprivation indices (continuous), education (college/university degree or others), smoking status (current/former or never), physical activity level (above moderate/vigorous/walking recommendation or not), general health status (excellent/good or fair/poor) and comorbidities. Comorbidities included obesity, diabetes mellitus, hypertension, history of heart diseases and history of stroke. Obesity was defined as body mass index (BMI) > 30 kg/m^2^, calculated as body weight in kilograms divided by height in meters squared. Diabetes mellitus was classified with any self-reported records, hospital diagnosis, use of anti-diabetic drugs or insulin, or serum glycosylated haemoglobin level of > 6.5%. Hypertension was defined as self-reported or hospital diagnosis or use of antihypertensive drugs records, or an average systolic blood pressure of  > 130 mmHg or an average diastolic blood pressure of  > 80 mmHg. History of heart diseases were determined as self-reported history of angina or heart attack.

### Statistical analyses

Continuous and categorical values were described as means and standard deviations (SDs) or numbers and percentages, respectively. For mortality analysis, cox proportional hazards regression models were used to estimate mortality risk for each 5-year increase in the OCT age gap. We further subdivided participants into three groups based on OCT age gaps compared to MAE, consistent with a previous study [26]. This will identify participants with an OCT-predicted age that deviates more than the MAE from the chronological age. We set the OCT age gap within the MAE ranges as the reference group to investigate associations OCT age with mortality risk. The results were adjusted for baseline age, sex, ethnicity, and townsend deprivation indices (model I); additional educational level, obesity, smoking status, physical activity level, diabetes mellitus, hypertension, history of heart diseases, and history of stroke (model II) in the Cox models. All variables met the proportional hazards assumptions. We also added age-square into the Cox models as sensitivity analysis.

A two-sided *p* value of < 0.05 indicated statistical significance. Analyses were performed using R (version 3.3.0, R Foundation for Statistical Computing, www.R-project.org, Vienna, Austria) and Stata (version 13, StataCorp, Texas, USA).

## Results

### OCT age accurately predicted chronological age

**Fig. 1:**
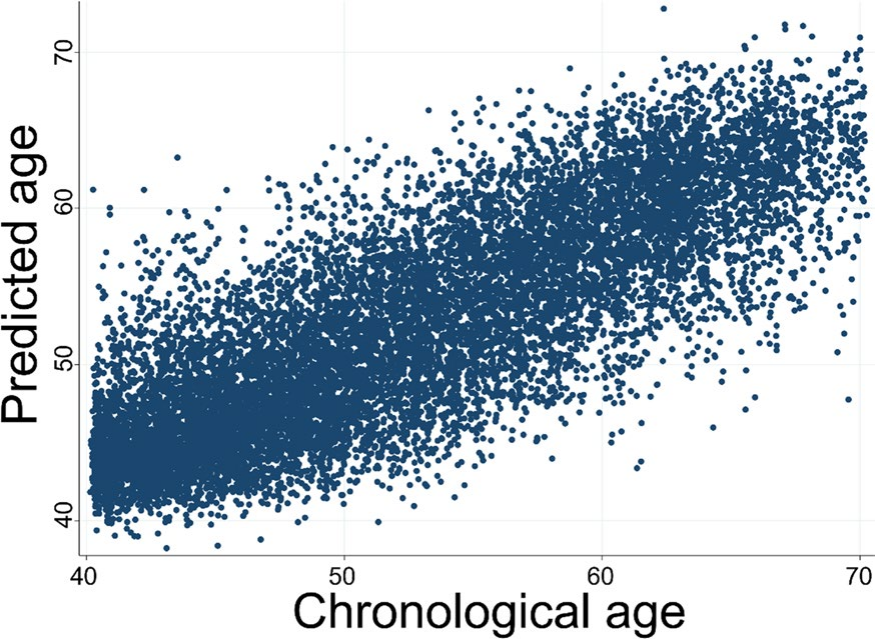
Performance of the Deep neural network model in relatively healthy participants without any reported diseases

The performance of the age prediction model in the testing dataset is illustrated in Fig. 1. The OCT age predicted by the DNN model significantly correlated with chronological age (r = 0.85). This model achieved a MAE of 3.27 years over the chronological age in the testing dataset. The MAEs of the OCT age prediction model in subjects aged < 55 years and > 55 years were 3.01 and 4.43, respectively.

### OCT age gap

Table 1 describes the baseline characteristics of study participants for mortality risk analysis. Among the 44,618 participants, 46.5% were male and 90.4% were white ethnicity with mean (SD) ages of 57.4 ± 7.94 years.

**Table 1:** Baseline characteristics of study participants for mortality risk analysis stratified by OCT age groups

Baseline Characteristics	Overall	OCT age gap			*P* value
		± 4 years	> 4 years younger	> 4 years older	
*N*	44,618	21,314	12,847	10,457	-
Death, *N* (%)	2,429	1,484(5.53)	640(6.48)	305(3.87)	**< 0.001**
Age, mean (SD), years	57.4(7.94)	57.4 (7.97)	61.0 (6.39)	52.9 (7.31)	** < 0.001**
Gender, *N* (%)					
Female	23,888 (53.5)	11,636 (54.6)	6,235 (48.5)	6,017 (57.5)	** < 0.001**
Male	20,730 (46.5)	9,678 (45.4)	6,612 (51.5)	4,440 (42.5)	
Ethnicity, *N* (%)					
White	40,342 (90.4)	19,365 (90.9)	11,805 (91.9)	9,172 (87.7)	** < 0.001**
Others	4,276(9.58)	1,949 (9.14)	1,042 (8.11)	1,285 (12.3)	
Deprivation index, mean (SD)	-1.02(2.99)	-1.02 (2.97)	-1.27 (2.91)	-0.72 (3.10)	** < 0.001**
Education level, *N* (%)					
College/university	15,226 (34.1)	7,242 (34.0)	3,853 (30.0)	4,131 (39.5)	** < 0.001**
Others	29,392 (65.9)	14,072 (66.0)	8,994 (70.0)	6,326 (60.5)	
Smoking status, *N* (%)					
Never	23,972 (54.1)	11,401 (53.8)	6,736 (52.8)	5,835 (56.1)	** < 0.001**
Former/current	20,375 (45.9)	9,789 (46.2)	6,023 (47.2)	4,563 (43.9)	
Drinking status, *N* (%)					
Never	2,099 (4.72)	996 (4.69)	627 (4.90)	476 (4.57)	0.485
Former/current	42,349 (95.3)	20,239 (95.3)	12,175 (95.1)	9,935 (95.4)	
Meeting PA recommendation, *N* (%)					
No	6,514 (17.9)	3,102 (17.9)	1742 (16.7)	1670 (19.5)	** < 0.001**
Yes	29,805 (82.1)	14,224 (82.1)	8,678 (83.3)	6,903 (80.5)	
Obesity, *N* (%)					
No	32,974 (74.3)	15,769 (74.3)	9,641 (75.5)	7,564 (72.7)	** < 0.001**
Yes	11,413 (25.7)	5,444 (25.7)	3,133 (24.5)	2,836 (27.3)	
History of diabetes, *N* (%)					
No	41,456 (92.9)	19,857 (93.2)	11,822 (92.0)	9,777 (93.5)	** < 0.001**
Yes	3,162 (7.09)	1,457 (6.84)	1,025 (7.98)	680 (6.50)	
History of hypertension, *N* (%)					
No	10,658 (23.9)	5,013 (23.5)	2,585 (20.1)	3,060 (29.3)	** < 0.001**
Yes	33,960 (76.1)	16,301 (76.5)	10,262 (79.9)	7,397 (70.7)	
History of heart diseases, *N* (%)					
No	42,674 (95.6)	20,416 (95.8)	12,089 (94.1)	10,169 (97.3)	** < 0.001**
Yes	1,944 (4.36)	898 (4.21)	758 (5.90)	288 (2.75)	
History of stroke, *N* (%)					
No	43,928 (98.4)	20,992 (98.5)	12,607 (98.1)	10,329 (98.8)	** < 0.001**
Yes	690 (1.55)	322 (1.51)	240 (1.87)	128 (1.22)	

*SD* standard deviation; Bold indicates *P* < 0.05

As the MAE of the OCT age prediction accuracy was 3.27 years, the cut-off value of age gap was set at 4 years to minimize the impacts from technical errors in age prediction. We divided the participants into three groups of patients who had an OCT age > 4 years smaller than the chronological age (> 4 years younger), OCT age within a range of 4 years from their chronological age (within ± 4 years), and OCT age > 4 years greater than the chronological age (> 4 years older). As shown in Table 1, the participants in > 4 years older OCT age groups were younger, and more likely to be female, non-white ethnicity, lower deprivation index, better education level, non-smokers, lower physical activity level, obese, without a history of diabetes, hypertension, heart diseases and stroke (all *P* < 0.001).

### OCT age gap and mortality

After a median follow-up of 11.0 years (IQR:10.9–11.1 years), 2,429 deaths (5.44%) were recorded. After adjusting for age, sex, ethnicity and deprivation, for each 5-year increase in OCT age gap, there was an 8% increased mortality risk (hazard ratio [HR] = 1.08, 95% confidence interval [CI]: 1.03–1.12, *P* = 0.001; Table 2). This was also significant in fully adjusted model (HR = 1.08, CI:1.02–1.13, *P* = 0.004).

**Table 2:** OCT age gap was associated with mortality risk using Cox proportional hazards regression model

		Model I		Model II	
OCT age gap	N	HR (95% CI)	P value	HR (95% CI)	P value
OCT age gap per five years	44,618	1.08 (1.03–1.12)	**0.001**	1.08 (1.02–1.13)	**0.004**
OCT age group					
> 4 years younger	12,847	0.86 (0.78–0.94)	**0.001**	0.84 (0.75–0.94)	**0.002**
± 4 years	21,314	1[reference]	**-**	1[reference]	**-**
> 4 years older	10,457	1.21 (1.06–1.37)	**0.004**	1.18 (1.02–1.37)	**0.026**

*HR* hazard ratio, *CI* confidence interval

Model I adjusted for age, sex, ethnicity and townsend

Model II adjusted for covariates in Model I + educational level, obesity, smoking status, physical activity level, diabetes mellitus, hypertension, history of heart diseases, and history of stroke

**Fig. 2:**
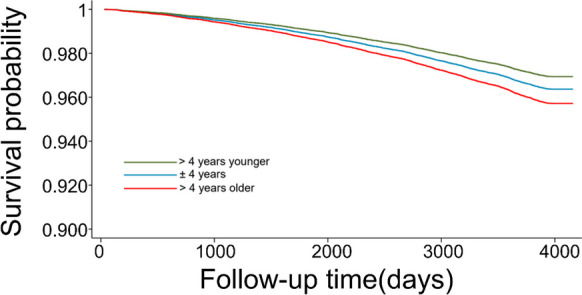
Adjusted survival curves for mortality risk by three OCT age groups. These survival curves adjusted for age, sex, ethnicity and townsend, educational level, obesity, smoking status, physical activity level, diabetes mellitus, hypertension, history of heart diseases, and history of stroke. Participants with an OCT age gap less than minus 4 years demonstrated a higher survival rate while OCT age gap more than 4 years demonstrated a poorer survival rate

Compared with groups of OCT age gap within ± 4 years, OCT age gap less than minus 4 years was associated with a 16% decreased mortality risk (HR = 0.84, CI: 0.75–0.94, *P* = 0.002) while OCT age gap more than 4 years showed an 18% increased risk of death incidence (HR = 1.18, CI: 1.02–1.37, *P* = 0.026). Similar results were observed with subdivided groups based on a cut-off value of 3.27 years (Supplementary Table 1). Fully adjusted survival curves for mortality risk by three OCT age groups are illustrated in Fig. 2. We further added age squared in the confounders of Cox regression model and similar associations between OCT age gaps and mortality were noted (Supplementary table 2). Subgroup analysis of different age groups are outlined in Supplementary Table 3.

## Discussion

In this study, we developed an OCT-based ageing biomarker using a DNN algorithm in a healthy population. The OCT age gap (the difference between OCT-predicted and chronological age) less than minus 4 years were associated with a lower risk of mortality while OCT age gaps of more than 4 years indicated an increased risk of mortality. These findings suggested that the OCT age gap, deviation from normal ageing, could predict mortality risk during a follow up of 11 years.

The OCT age achieved an excellent performance in biological age prediction compared with previously well-established ageing biomarkers where a smaller MAE indicates a higher accuracy. The OCT age achieved a MAE of 3.27 years which outperformed retinal age of 3.5 years, [6] epigenetic clock of 3.3–5.2 years, [27, 28] brain age of 4.3–7.3 years, [24, 29] transcriptome age of 6.2–7.8 years, [30, 31] and blood profiles-based age of 5.5–5.9 [32, 33]. Moreover, the OCT age derived from retinal OCT imaging is non-invasive and quick while previous markers are more invasive, costly and/or time consuming.

To the best of our knowledge, our study is the first to develop a biological age based on OCT images and the first to directly correlate OCT age gap, difference between OCT age and chronological age, with mortality risk. Compared to the recently developed retinal age from fundus images, OCT age achieved an accurate performance of age prediction by capturing 3D cross-sectional, neuroanatomical and vascular changes in the retina at a high resolution. Furthermore, our study provided a proposed approach to define cut-off value of accelerated ageing around the mean absolute error (MAE) while it remained unknown how much retinal age deviated from normal ageing considered as accelerated ageing. A well-defined cut-off value determined the threshold between accelerated and normal ageing, allowing healthcare professionals to interpret test results effectively.

Our study showed that an OCT-predicted age higher than chronological age was associated with an increased mortality risk, and conversely, an OCT age lower than the chronological age a reduced mortality risk. Emerging evidence is growing to support that OCT parameters have been associated with age-related diseases. For example, the retinal nerve fiber layer (RNFL) thinning was significantly associated with age-related diseases including glaucoma, [34] Parkinson’s disease [35], and Alzheimer's disease [36]. Retinal macular thickness was strongly associated with systemic hypertension and cardiovascular diseases. [37–39]

Although the biological mechanisms underlying the OCT ageing association have been not fully established, several hypotheses have been proposed. It has been suggested that ageing could induce oxidative stress, chronic inflammation, DNA damage,[40] leading to retinal ganglion cell and axonal loss [41, 42], presented as RNFL thinning captured by OCT [43, 44]. Moreover, major blood vessels thinning with ageing is another possible explanation for the association between OCT age and mortality risk [45, 46]. Preliminary evidence suggests that oxidative stress and endothelial dysfunction may underlie the adverse effects of ageing on the retinal vascular system [45, 47]. Further research is needed to elucidate the aging process.

Our findings have several important clinical implications. This study revealed information from the OCT images could be summarized as a single age index with a clear interpretation of biological age. This indicates that OCT age may provide a promising tool for personalized ageing quantification and tailored-risk stratification. Generally, individual aberrations from normal ageing would help individuals to be aware of their ageing status and take personalized health action and intervention. With OCT now becoming increasingly accessible in hospital and community settings, early detection of accelerated ageing and personalized intervention will have a significant positive impact on public health, particularly in the context of an aging global population. [48]

### Strength and limitations

To the best of our knowledge, this was the first study to investigate OCT age gap prediction of mortality risk. The strengths of the current study included its large sample size, multicenter study design, standardized protocols in acquiring OCT images, extensive adjustments for covariates and long follow-up. Despite this, some limitations should be acknowledged. The study population was from the UK and participants were mostly Caucasian, young, and healthy, which may limit the external generalizability [49]. Due to the observational study design, we could not infer causation.

## Conclusion

We developed an accurate ageing biomarker from OCT images using DNN models. The OCT age gap, defined as the difference between OCT-predicted age and chronological age was associated with future mortality risk. This suggests that the OCT age gap can be used as a biomarker of mortality.

### Supplementary Information

Below is the link to the electronic supplementary material.

Supplementary file1 (DOCX 28.8 KB)

## Data Availability

The data that support the findings of this study are publicly available in https://www.ukbiobank.ac.uk/ via reasonable application.
