# Supplementary material for: Deep neural network-estimated age using optical coherence tomography predicts mortality
Source: GeroScience. 2023 Sep 21;46(2):1703–11. doi: 10.1007/s11357-023-00920-4 (PMC10828229; doi:10.1007/s11357-023-00920-4)
Supplement: Supplementary file 1 — Supplementary file1 (DOCX 28.8 KB) [file 11357_2023_920_MOESM1_ESM.docx]

Supplementary Table 1. OCT age gaps was associated with mortality risk using 3.27 years as the cut-off value

|  |  | **Model I** | | **Model II** | |
| --- | --- | --- | --- | --- | --- |
| **OCT age** | **N** | **HR (95% CI)** | **P value** | **HR (95% CI)** | **P value** |
| OCT age group |  |  |  |  |  |
| > 3.27 years younger | 14,873 | 0.90 (0.82-0.99) | **0.023** | 0.89 (0.80-0.99) | **0.028** |
| +3.27 years | 21,314 | 1[reference] | **-** | 1[reference] | **-** |
| > 3.27 years older | 14,872 | 1.19 (1.06-1.34) | **0.005** | 1.16 (1.01-1.33) | **0.035** |
| HR = hazard ratio; CI = confidence interval. | | |  |  |  |
| Model I adjusted for age, sex, ethnicity and townsend. | | | |  |  |
| Model II adjusted for covariates in Model I + educational level, obesity, smoking status, physical activity level, diabetes mellitus, hypertension, history of heart diseases, and history of stroke. | | | | | |

Supplementary Table 2. Sensitivity analysis for associations between OCT age gaps and mortality risk.

|  |  | **Model III** | | **Model IV** | |
| --- | --- | --- | --- | --- | --- |
| **OCT age** | **N** | **HR (95% CI)** | **P value** | **HR (95% CI)** | **P value** |
| OCT age gap per five years | 44,618 | 1.08 (1.03-1.13) | **0.001** | 1.08 (1.03-1.14) | **0.003** |
| OCT age group |  |  |  |  |  |
| > 4 years younger | 14,873 | 0.85 (0.78-0.94) | **0.001** | 0.84 (0.75-0.93) | **0.001** |
| + 4 years | 21,314 | 1[reference] | **-** | 1[reference] | **-** |
| > 4 years older | 14,872 | 1.21 (1.06-1.38) | **0.004** | 1.19 (1.03-1.38) | **0.022** |
| HR = hazard ratio; CI = confidence interval. | | |  |  |  |
| Model III adjusted for age, age squared, sex, ethnicity and Townsend. | | | |  |  |
| Model IV adjusted for covariates in Model I + educational level, obesity, smoking status, physical activity level, diabetes mellitus, hypertension, history of heart diseases, and history of stroke. | | | | | |

Supplementary Table 3. Subgroup analysis for associations between OCT age gaps and mortality risk.

|  | **Age <55 years** | | | **Age >55 years** | | | |
| --- | --- | --- | --- | --- | --- | --- | --- |
| **OCT age** | **HR (95% CI)** | **P value** | | **HR (95% CI)** | | **P value** | |
| OCT age gap per 5 years | 1.14 (1.00-1.31) | **0.047** | | 1.07 (1.01-1.13) | | **0.017** | |
| OCT age group |  |  | |  | |  | |
| > 4 years younger | 0.63 (0.39-1.03) | 0.067 | | 0.85 (0.76-0.95) | | **0.004** | |
| + 4 years | 1[reference] | **-** | | 1[reference] | | **-** | |
| > 4 years older | 1.32 (1.00-1.74) | 0.051 | | 1.12 (0.94-1.35) | | 0.199 | |
| HR = hazard ratio; CI = confidence interval. | | |  | |  | |  |
| Model adjusted for age, ethnicity, gender, townsend, educational level, obesity, smoking status, physical activity level, diabetes mellitus, hypertension, history of heart diseases, and history of stroke. | | | | | | | |
